# Successful side-viewing endoscopic hemoclipping for Dieulafoy-like lesion at the brim of a periampullary diverticulum

**DOI:** 10.1186/1471-230X-10-24

**Published:** 2010-02-23

**Authors:** Wan Sik Lee, Sung Bum Cho, Sun Young Park, Change Hwan Park, Young Eun Joo, Hyun Soo Kim, Sung Kyu Choi, Jong Sun Rew

**Affiliations:** 1 LDepartment of Internal Medicine, Division of Gastroenterology, Chonnam Medeical University Hwasun Hospital, Hwasun, Jeonnam, South Korea

## Abstract

**Background:**

Duodenal Dieulafoy's lesions are rare and only several cases were reported so far. Their characteristic appearance and location make it difficult to be diagnosed in the clinical practice. Massive bleeding often results from these lesions and can impede the accurate early treatment.

**Case presentation:**

67 years old male patient suffered a fatal bleeding from Dieulafoy-like lesion located at the mouth of the periampullary diverticulum. Inintial endoscopic therapy and radiologic embolization failed to stop the bleeding, while direct observation and hemoclipping by the side viewing endoscopy successfully established correct diagnosis and permanent cure of the lesion.

**Conclusion:**

Aggressive endoscopic examinations combined with the accurate endoscopic threatment should be adopted when Dieulafoy-like lesion is suspected as a possible cause of the proximal small bowel hemorrahge. Verification of the diagnosis and definitive treatment often needed repeated examination by side-viewing endoscope as well as stabilization of the patient.

## Introduction

Dieulafoy lesions are an arteriovenous malformation typically found in the gastrointestinal tract and an uncommon cause of gastrointestinal bleeding[1]. This lesion comprises small pea sized lesion appearing as a mucosal defect with an artery protruding from its base. 75 to 95% of the cases were found in the stomach. Extragastric lesions are relatively rare and large bowel was more frequent location than small bowel[2]. Hemorrhage from this lesions are often torrential and life threatening.

Periampullay diverticula are discovered incidentally in patients during endoscopic retrograde cholangiopancreatography, are usually asymptomatic, but can be the source of significant morbidity mostly due to the pancreatobiliary complications[3]. Generally, Duodenal diverticular seldom cause hemorrhage, but when they do bleed, it is difficult to make diagnosis at time, hence, they are often fatal. In addition, experience with the endoscopic management of such bleeding is limited as compared to the colonic diverticular bleeding. In earlier reports, endoscopic treatment was used as a only temporary measure before the elective operation[4]. In the case reported here, hemoclip placement through side-viewing duodenoscope was a definitive therapy for the duodenal diverticular bleeding caused by Dieulafoy lesion.

## Case Reports

67 years old male patient was admitted presenting massive hematemesis and melenic stool in two days. He had suffered a blunt abdominal trauma from traffic accident 1 month ago and given analgesics for 12 days during hospitalization. He had no previous history of other health problems. He was anemic and tachypneic and systolic blood pressure was 90 mmHg. Laboratory data was as follows; Hemoglobin 5.5 g/dL, Platelet 44 × 103/mm, Albumin 1.7 g/dL, Prothrombin time 14.6 second (INR 1.26), AST 20 IU/L, ALT 12 IU/L, BUN 43.7 mg/dL, Creatinine 0.6 mg/dL.

**Figure 1:**
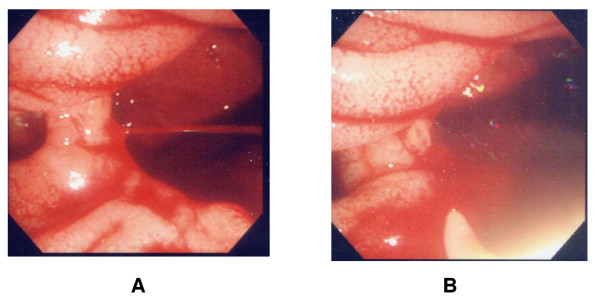
**Initial manifestation and treatment**. A. On duodenoscopic exam, at the margin of large(about 6 cm) periampullary diverticulum was a small exposed vascular turft emanating the spurting jet of blood. B. Epinephrine and saline mixture was injected to lessen the bleeding, but complete hemostasis was not achieved.

**Figure 2:**
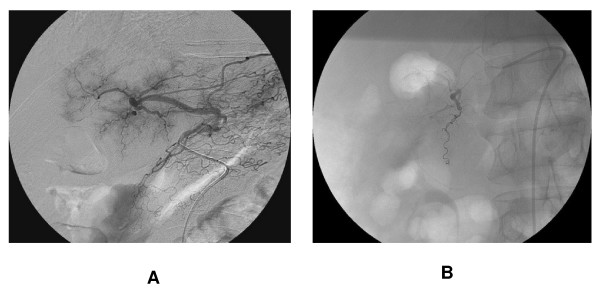
**Radiologic intervention**. A. On celiac axis angiogram, a extravasation of the contrast media from the distal branch of gastroduodenal artery was noted. B. The branch was superselected and embolized with microcoils.

Urgent endoscopic examination(XQ-240; Olympus, Japan) revealed large clot and fresh blood at the descending portion of the duodenum but exact bleeding focus could not be found. Melena continued and the patient's condition deteriorated. On side-viewing duodenoscopy(TJF-240; Olympus, Japan) performed next day, spurting jet of blood was observed from the small vascular turft located at the margin of the large(about 6 cm in size) periampullary diverticulum (Figure 1). After Epinephrine and saline mixture (about 30 cc) was injected maginally to lessen the bleeding, angiography was tried to intervene the bleeding vessel directly. On celiac axis angiogram, there was a extravasation of contrast media from distal branch of gastroduodenal artery (Figure 2A). This branch was superselected and embolized with the five microcoils (Figure 2B). Bleeding seemed to be temporarily controlled, however, the patient sustained another bout of severe hematemesis following day.

**Figure 3:**
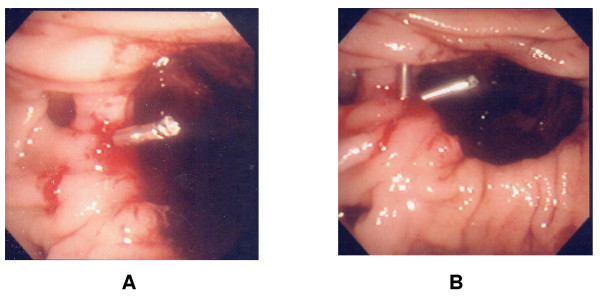
**Rebleeding and final endoscopic hemostasis**. A. After angiographic intervention failed, duodenoscopic examination revealed constant bleeding B. Two metalic hemoclips were successfully placed through the side-viewing endoscope at the Dieulafoy-like lesion and instant hemostasis was achieved.

Another session of duodenoscopic exam demonstrated the still bleeding Dieulafoy like lesion at the same focus. Though it was difficult to locate the delivery catheter over the bleeding site, two hemoclips (HX-600;Olympus) were applied directly at the lesion through the working channel of the side-viewing duodenoscope with the use of an application device(HX-5LR; Olympus) (Figure 3). Bleeding cessed immediately. The patient recovered dramatically from the shock status.

**Figure 4:**
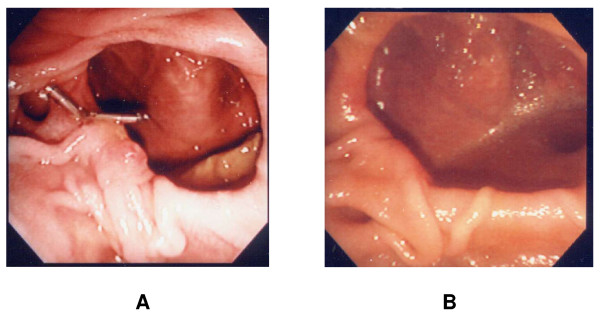
**Short and long term outcome**. A. 3 days later, hemoclips were seen securely placed at the lesion. No recurrent bleeding occurred. B. 2 months later, hemoclips were gotten rid of spontaneously leaving only faint scar.

3 days later, hemoclips were seen securely placed at the brim of the periampullary diverticulum (Figure 4A). No recurrent bleeding occurred. On follow up examination performed 2 months later, hemoclips was detached spontaneously, and the lesion was completely healed without leaving any sequels (Figure 4B).

## Discussion

While gastric Dieulafoy's lesions are well known, there was little experience about the Dieulafoy lesions located at small bowel. In the gastric lesion, endoscopic treatment is regarded as a initial therapy of choice. For the duodenal lesion, albeit in a small cases, endoscopic treatment largely replaced the need of surgery[5]. This fact, combined with the advent of the effective endoscopic hemostatic methods, could explained the difficulty to obtain a surgical specimen for definitive pathologic diagnosis. Therefore, some authors suggest the Dieulafoy-like lesion is a more accurate description when only endoscopic observation is available. Furthermore, repeated endoscopic examination is frequently necessary because the bleeding point can be so small that unless there was a ongoing bleeding, it may be difficult to detect the lesion.

In our case, the lesion appeared as a active arterial spurting of blood without accompanying ulceration and through normal surrounding mucosa. This fulfills the commonly agreed endoscopic criteria for the diagnosis of Dieulafoy lesion. Forward viewing conventional endoscopy fails to locate the lesion because the lesion is at the perimapullary area. The precise location of the lesion was a inlet of the periampullary diverticulum about 2 cm from the ampullar opening.

Duodenal diverticulum are found mostly from the concave aspect of the duodenum. In one study, 62% were in the second part and 30% in the third part and 8% in fourth part of the duodenum[6]. They are mostly asymptomatic, but bleeding, inflammation and perforation are the rare complication. Periampullary diverticulum refers to the diverticula within 2.5 cm of the ampulla of Vater. Although periampullary diverticula are rarely symptomatic, their association with gallstones and pancreatitis is well documented[7].

Actual bleeding site in our case can not be attributed solely to the diverticular bleeding. In a diverticular bleeding, usual bleeding site is in the dome of diverticulum where aberrant blood vessels lay over the thin walled luminal surface[4,8]. We observe the bleeding spot at the marginal surface of the diverticular inlet. Hence, It is reasonable to think that actual bleeding is from the Dieulafoy-like lesion at the brim of diverticulm. But it is still possible that this lesion is in fact due to the development of a Dieulafoy-like lesion as a result of a mechanical or other stresses to the mucosal or submucosal vessels at the mouth of the diverticulum. Recent use of non-steroidal anti-inflammatory drugs can also be regarded as a contributing factor in our case.

There were many endoscopic therapeutic options for the Dieulafoy bleeding. Endoscopic therapy have been very useful for the treatment of Dieulafoy lesions, especially when they were detected and able to be reached by endoscope. Endoscopic therapy includes epinephrine includes monotherapy (epinephrine, sclerosant, alcohol, thermal probe) [9-11] or combination therapy(injection followed by thermal probe coagulation)[2,12] or mechanical hemostasis methods(band ligation, hemoclip)[13,14].

There was report about the successful hemochip placement at the Dieulafoy-like lesion in a duodenal diverticulum through a forward-viewing endoscope[15]. But, conventional forward-viewing endoscope could not yield a correct diagnosis particularily due to the difficult location of the lesion. The medial and marginal portion of duodenal diverticulum can only be inspected by the side-viewing endoscope. Any maneuvers using the catheter through a side-viewing endoscope were considered difficult because of acute angulation at the tip of the scope. After hemostasis was not achieved by epinephrine injection, we could not consider any endoscopic therapeutic options other than angiographic intervention, because rather limited view of field and motion ability of the side-viewing endoscope hinder any further therapeutic maneuvers. But when the rebleeding occurred, after a failure of angiographic embolization, We tried the mechanical hemostasis methods by using metal hemoclips. Though it was difficult to locate and release the hemoclips at the time, immediate hemostasis achieved instantly after hemoclip application. Thus the patient recovered uneventfully without rebleeding while avoiding surgery that could cause unpredictable morbidities.

## Conclusions

An high index of suspicion about the Dieulafoy leisons should be given to the case of upper gastrointestinal bleeding when more common causes have been ruled out by routine endoscopy. Aggressive but careful endoscopic examinations combined with the accurate endoscopic threatment using adequate therapeutic methods may help clinicians diagnose and treat many if not most of the Dieulafoy lesions. Periampullary or duodenal diverticular lesions should always be sought by the side-viewing endoscope when the bleeding is from the descending duodenum and conventional endoscope can not verify the causes. Although it tend to be not easy to perform, hemoclip placement through a side-viewing endoscope can be effective hemostatic method in this setting.

## Competing interests

The research was supported by a grant from the institute of Medical System Engineering (iMSE) in the GIST, Korea.

## Authors' contributions

SBC, SYP, CHP, YEJ made substantial contributions to the conception and interpretation of clinical data and case-related studies, and clinical decisions. HSK, SKC, JSR participated in its design and coordination and help to draft the manuscript. All authors read and approve the final manuscript.

## Pre-publication history

The pre-publication history for this paper can be accessed here:

http://www.biomedcentral.com/1471-230X/10/24/prepub
